# Defining the oral microbiome by whole-genome sequencing and resistome analysis: the complexity of the healthy picture

**DOI:** 10.1186/s12866-020-01801-y

**Published:** 2020-05-18

**Authors:** Elisabetta Caselli, Chiara Fabbri, Maria D’Accolti, Irene Soffritti, Cristian Bassi, Sante Mazzacane, Maurizio Franchi

**Affiliations:** 1 Section of Microbiology and Medical Genetics, Department of Chemical and Pharmaceutical Sciences, University of Ferrara, Ferrara, Italy; 2 CIAS Research Center, University of Ferrara, Ferrara, Italy; 3 Section of Dentistry, Department of Biomedical and Specialty Surgical Sciences, University of Ferrara, Ferrara, Italy; 4 NGS Service, Department of Morphology, Surgery and Experimental Medicine, University of Ferrara, Ferrara, Italy

**Keywords:** Oral microbiome, Whole-genome sequencing (WGS), Site-specific microbiome map, Resistome

## Abstract

**Background:**

The microbiome of the oral cavity is the second-largest and diverse microbiota after the gut, harboring over 700 species of bacteria and including also fungi, viruses, and protozoa. With its diverse niches, the oral cavity is a very complex environment, where different microbes preferentially colonize different habitats. Recent data indicate that the oral microbiome has essential functions in maintaining oral and systemic health, and the emergence of 16S rRNA gene next-generation sequencing (NGS) has greatly contributed to revealing the complexity of its bacterial component. However, a detailed site-specific map of oral microorganisms (including also eukaryotes and viruses) and their relative abundance is still missing. Here, we aimed to obtain a comprehensive view of the healthy oral microbiome (HOM), including its drug-resistance features.

**Results:**

The oral microbiome of twenty healthy subjects was analyzed by whole-genome sequencing (WGS) and real-time quantitative PCR microarray. Sampled oral micro-habitat included tongue dorsum, hard palate, buccal mucosa, keratinized gingiva, supragingival and subgingival plaque, and saliva with or without rinsing. Each sampled oral niche evidenced a different microbial community, including bacteria, fungi, and viruses. Alpha-diversity evidenced significant differences among the different sampled sites (*p* < 0.0001) but not among the enrolled subjects (*p* = 0.876), strengthening the notion of a recognizable HOM. Of note, oral rinse microbiome was more representative of the whole site-specific microbiomes, compared with that of saliva. Interestingly, HOM resistome included highly prevalent genes conferring resistance to macrolide, lincosamides, streptogramin, and tetracycline.

**Conclusions:**

The data obtained in 20 subjects by WGS and microarray analysis provide for the first time a comprehensive view of HOM and its resistome, contributing to a deeper understanding of the composition of oral microbiome in the healthy subject, and providing an important reference for future studies, allowing to identify microbial signatures related to functional and metabolic alterations associated with diseases, potentially useful for targeted therapies and precision medicine.

## Background

The microbial community of the oral cavity is the second most complex of the human body, after the gut-associated microbiome.

The oral cavity is constantly exposed to both the inhaled and ingested microbes, with more than 700 species comprehensive of bacteria, fungi, viruses, archaea, and protozoans [1]: only 54% of these species is cultivable and identified, the 14% cultivable but not identified, the 32% not even cultivated [2].

The oral microbiome composition differs between the various micro-habitat and each individual has its “microbial identity” consisting of its own and unique microbial population [3]. Although these inter-individual differences, the principal function of the microbiome is the same in every person [4]. The microbial population has an active role in the physiological, nutritional and defensive development of each individual. The commensal microbiome has an important role in the maintenance of oral and systemic health: its delicate balance can be easily altered, causing oral pathologies such as cavities endodontic disease, periodontal diseases, osteitis and tonsillitis [5–7] and can be associated with the development of several systemic diseases, for example cardiovascular disease [8], ictus [9], pre-term childbirth [10], diabetes [11], pneumonia [12], obesity [13, 14], colon carcinoma [15] and psychiatric issues [16].

Studies involving healthy participants are therefore crucial do define properly the human oral microbiota in health before trying to confirm any correlation of the oral microbiome with peculiar disease conditions. The microbiome of the oral cavity is extraordinarily complex since each different surface of the oral cavity offers a unique ecologic niche, with its nutrient and environmental conditions, suitable for only a certain number of microbial organisms [17, 18]. Consequently, each micro-habitat has to be sampled with adequate equipment. For example, for oral mucosa, the use of sterile brushes [19] and nylon sterile microbrushes [20] have been reported. Sterile Gracey curettes are usually used for plaque sampling on teeth hard tissues [21–23]. Other devices included are endodontics paper cones [24], sterile microbrushes [20], sterile toothpicks [25, 26], and floss [20, 26]. For saliva sampling, the majority of studies analyze the non-stimulated saliva [27], but other studies rather used oral rinse (saliva after rinsing), to obtain a higher fraction of microbes possibly adhered to oral surfaces [28]. The Human Microbiome Project (HMP) codifies a non-invasive protocol for sampling the oral cavity microbiome, adopted in several investigations [21–23].

In the past, studies on the oral microbiome were hampered in the past by the limitations of the conventional culture-dependent techniques used, as many species of the abundant oral microflora are not cultivable. More recently, the new molecular techniques based on next-generation sequencing (NGS) of the 16 rRNA gene of the bacterial genome, allowed to evidence the complexity of the bacterial component of the oral microbiome. In recent years, NGS has, in fact, represented the standard for studying the composition of microbial communities, allowing to differentiate bacteria by sequencing the variable regions of the gene coding for the 16S ribosomal RNA (rRNA). Such a method greatly improved our knowledge of the bacterial component of the oral microbiome, as it does not depend on culture isolation and permits to evidence also unsearched bacteria [2, 29]. However, while it allows identification of bacteria at the level of species, this method does not usually provide sufficient information to resolve communities at the sub-species level, nor it can detect eukaryotic microorganisms and/or viruses. Instead, the species-level resolution obtained by NGS is not adequate for transmission studies or for exploring subspecies variation in disease association, and the oral microbiome includes also important non-bacterial components, including eukaryotic microbes (fungi, protozoa) and viruses.

The reports on a normal microbiome, however, have been almost exclusively restricted to the bacteriome, and there are very few reports on the mycobiome–fungal microbiome and on other microorganisms. Fungi have been reported very often as members of the healthy oral microbiota, where up to 101 species have been described [28, 30], including *Candida* spp., followed by *Cladosporium*, *Aureobasidium*, *Saccharomyces*, *Aspergillus*, *Fusarium*, and *Cryptococcus* species. Archaea have also been detected, although they represent a minor part and are generally elevated in subjects with periodontitis [31–33]. Among protozoa, *Entamoeba gingivalis* and *Trichomonas tenax* are the most commonly found protozoa and are mainly saprophytic [34]. When present, viruses have been often correlated to diseases, such as for herpesviruses, papillomaviruses or HIV.

To assess simultaneously the presence and amount of all the microbial components potentially present in the oral cavity, the Whole Genome Sequencing (WGS) was introduced very recently, although quite sporadically and in a relatively low number of samples from the oral cavity [23, 35, 36]. Whole metagenome shotgun sequencing can, in fact, provide strain-level data, although it has been considered unsuitable for large-scale studies, as achieving sufficient depth of sequencing can be cost-prohibitive, and even with adequate coverage, deconvoluting complex communities such as the oral microbiota is computationally very challenging.

Thus, there is a need for studies providing a high-resolution high-throughput characterization of the healthy microbial communities in the oral microhabitats.

Besides, an important aspect that was not addressed in depth in the studies available so far is related to the antimicrobial resistance (AMR) features of the oral microorganisms, since very few reports are available on the resistome of the healthy oral microbiome [37–39]. On the contrary, based on the growing AMR concern, it would be useful to have data on the prevalence and type of drug resistance of the microbes composing the healthy oral microbiome, which might be very easily acquired and transmitted through aerosol and contact.

Thus, the present study aimed to obtain a site-specific map of the oral microbiome of the young adult healthy subject, using WGS to characterize the peculiar microbial population of each oral micro-habitat and evaluating the representativeness of saliva and saliva after rinsing to describe the whole oral microbiome. The resistome of the healthy microbiome of the oral cavity was also assessed, by real-time quantitative PCR (qPCR) microarray.

The findings provide for the first time a complete critical baseline for future studies interpreting microbiome-related diseases. The collected results also provided indications that microbiome in saliva after rinsing can give a picture representative of the site-specific samples, and might be therefore used for future comparative studies.

## Results

### Study population features

With the aim to obtain a site-specific map of the healthy oral microbiome (HOM), twenty healthy young adults were enrolled in the study following the inclusion criteria listed in Table 1.

**Table 1:** List of inclusion and exclusion criteria for subjects enrollment in the study

Inclusion	Exclusion
• Male and female subjects • Age 18-30 years • Good general health: free of systemic diseases such as diabetes, HIV infection or genetic disorder, ongoing malignant disease of any type that could interfere with the evaluation of the study objectives • Good oral health: free of oral pathologies such as leukoplakia, erythroplakia, oral lichen planus (OLP) • Availability for the 6-month duration of the study for an assigned subject • Signed Informed Consent Form	• Pregnant or lactating women • More than 8 missing teeth (with missing teeth, accounted for by third molar extractions, teeth extracted for orthodontic purposes, teeth extracted because of trauma, or congenitally missing teeth) • Presence of orthodontic appliances • Chronic dry mouth (clinically assessed) • Significant halitosis (clinically assessed) • Periodontitis • Untreated carious lesions or oral abscesses • Current or past (within 3 months before enrolment) assumption of medications that may influence oral microbiome (corticosteroids, calcium channel blockers, systemic antibiotics). • Non-surgical and/or surgical mechanical/manual periodontal debridement within 3 months before enrolment. • Heart diseases or blood pressure alteration that requires a medication • Renal, hepatic, gastrointestinal disease that requires a medication • Diabetes • Presence of any sexually transmitted disease (STD), HIV, HCV infection • Genetic disorders potentially interfering with the evaluation of the study objectives • Chronic obstructive pulmonary disease and asthma • Neoplastic lesions or paraneoplastic syndrome; tumors or significant pathology of the soft or hard tissues of the oral cavity (such as LPO, erythroplakia, leukoplakia, candidiasis) • Current radiotherapy or chemotherapy

Following these criteria twenty healthy subjects, including 10 males and 10 females, having a mean age corresponding to 24.7 years (range 21–30), were enrolled in the study. Each subject was also characterized for the number of teeth, the Plaque Score (O’Leary PL, which represents the percentage of sites, four per tooth, presenting plaque; mean value ± SD, 24.15 ± 8.54), the Bleeding on Probing (BOP) index (representing the percentage of sites, six per tooth, presenting bleeding on periodontal probing; the optimal value in the healthy subject should be less than 10%), the type of oral hygiene devices (OHD) used at home (manual or powered toothbrush, and use of interproximal devices, floss or interproximal brush). Besides, for female subjects, the use of oral contraceptives and the monthly period (out of a regular period of 4 weeks) were also registered to assess any eventual variation.

For BOP index, although the HMP protocol allows a slight degree of gingivitis, for the present study we maintained the exclusion criteria for BOP≥10%, following the definition of the periodontal healthy subject of the World Workshop 2017 of the American Academy of Periodontology and European Federation of Periodontology [40].

The demographic and clinically significant features of enrolled subjects are summarized in Table 2**.**

**Table 2:** Demographic and clinical features of the study population

Subject ID n.	Teeth n.	Plaque Score^a^	BOP^b^	OHD (Oral Hygiene Devices at home)	
				Toothbrush	Interproximal device
1	29	52,30	9,77	manual	none
2	29	24,14	9,77	powered	none
3	32	19,79	8,33	powered	floss
4	30	12,22	2,22	powered	none
5	31	22,62	1,79	manual	none
6	28	23,21	1,19	manual	floss
7	30	20,00	2,78	manual	none
8	32	28,65	1,56	powered	none
9	31	25,27	5,38	manual	none
10	30	27,78	2,22	powered	floss
11	28	17,26	2,97	powered	none
12	30	22,39	9,89	manual	floss
13	28	30,36	2,98	manual	floss
14	32	27,98	4,17	manual	floss
15	28	8,93	0,60	powered	none
16	32	20,31	1,04	manual	floss
17	31	29,03	2,15	manual	none
18	28	22,02	4,76	manual	floss
19	30	23,89	2,78	powered	floss
20	28	25,00	3,57	powered	none

^a^Plaque Score (O’Leary PL, which represents the percentage of sites, four per tooth, presenting plaque; the optimal value should be less than 20%),

^b^Bleeding on Probing (BOP) index (representing the percentage of sites, six per tooth, presenting bleeding on periodontal probing; the optimal value should be less than 10%)

Overall, the mean number of teeth was 29.85 (range 28–32), the Plaque Score mean value corresponded to 24.16% (range 8.93–52.3%) and the BOP index was largely below 10%, with a mean value of 3.99% (range 0.6–9.89%). Eleven subjects used manual toothbrushes, whereas 9/20 used a powered toothbrush. Nine subjects also used teeth floss in addition to the toothbrush, three of which combined with a powered toothbrush and six with manual brushing, whereas 11/20 did not use any interproximal hygiene device.

### Microbiome analysis

All the enrolled subjects were included in the study and analyzed for healthy oral microbiome composition. From each subject, eight different specimens were collected, including saliva, oral rinse (saliva after rinsing), four soft tissues sampled by sterile swabs (tongue, hard palate, buccal mucosa, keratinized gingiva), and two hard tissues sampled by sterile curettes (supra- and sub-gingival plaque).

For saliva collection, the subject was asked to let the saliva collect in the mouth for at least 1 min, then asked to drool it into a sterile 50 ml sterile collection tube, to collect a minimum volume of 2 ml. In the case of the collection of saliva after rinsing, the subject was asked to perform a mouth rinsing with 15 ml of sterile PBS, for 1 min, and then to drool the oral rinse into a 50 ml sterile collection tube. All specimens were put in sterile tubes, refrigerated immediately after collection, and processed within 1 hour from the collection.

Total DNA was extracted from each sample by a commercial kit with the addition of a pre-lysis step with 5 mg/ml of lysozyme to obtain optimal lysis of Gram-positive bacteria. Extracted DNA was quantified by spectrophotometric reading at 260/280 nm, and checked by a pan-bacterial (*panB*) PCR and a human β-actin PCRs, to verify that all extracted DNAs were amplifiable both for bacterial and eukaryotic genes.

Following DNA checking, 100 ng of each sample were analyzed by whole-genome sequencing (WGS) by the NGS Service (Department of Morphology, Surgery and Experimental Medicine, University of Ferrara). WGS libraries were prepared using NEBNext® Fast DNA Fragmentation and Library Prep Kit for Ion TorrentTM and sequenced by using the Ion Gene Studio S5 System.

Taxonomic assignment was performed using Kraken2 (Pubmed ID: 24580807) and a database consisting of archaea, bacteria, fungi, protozoa, and viruses. Raw data and protocols used for data elaboration have been uploaded by the NGS Service in the European Nucleotide Archive (ENA) website (accession number PRJEB36291). A threshold corresponding to 10 copies was considered for the positivity of each detected genus/species.

**Fig. 1:**
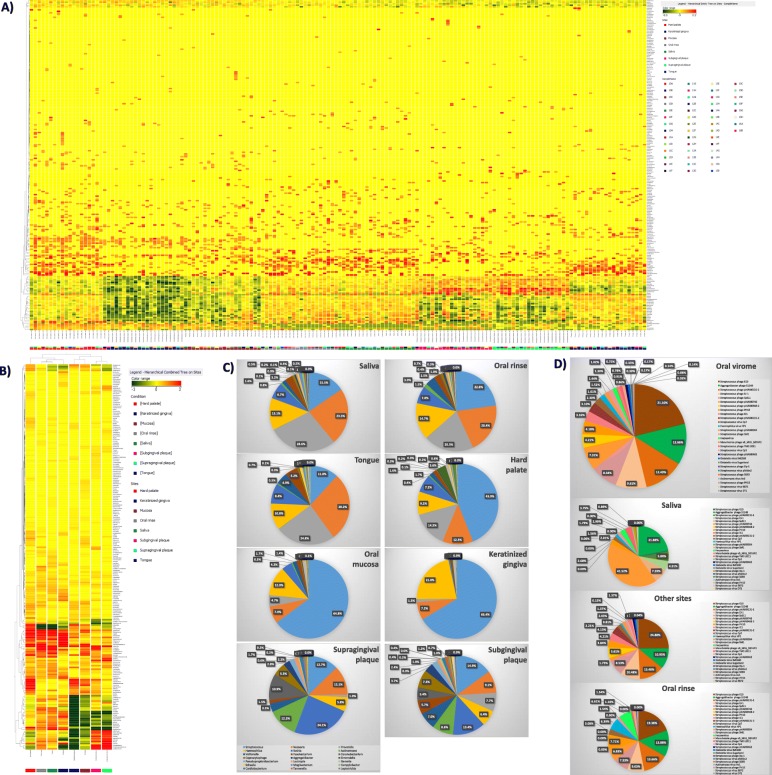
Relative abundance and distribution of the microbial genera detected in the oral cavity. **a** Heatmap representation of genera detected by WGS analysis in each sampled site from each enrolled subject. Hierarchical legends are also shown. **b** Heatmap representation of genera detected by WGS analysis in the clustered different sampled sites. Hierarchical legends are also shown. **c** Percentage distribution of detected genera in the different sampled sites of the oral cavity. **d** Prevalence of viruses detected in the oral cavity: upper panel, composition of the whole oral virome; lower panels, comparison between virus species prevalence detected in saliva, oral rinse, and site-specific sampled sites (other sites: hard and soft tissues). Results are expressed as the percentage of counts of each species on the total counts of the whole oral virome in all sampled sites

Overall, HOM analysis by WGS detected at least 218 microbial genera and 570 species, including bacteria, mycetes, and viruses. In particular, as shown in Fig. 1, the analysis showed that specimens from the same sampled site defined recognizable clusters, although the expected presence of inter-individual variability. By clustering the specimens derived from each specific sampled sites (including those representing saliva and oral rinse), the differences among oral microhabitats were particularly evident.

The comparison between the prevalence of the most abundant bacterial genera, in the different sampled oral sites (namely 42 genera representing at least 0.1% of total bacteria in at least one sampled site), showed a clear difference in microbial distribution among sites, as expected (Fig. 1c).

In particular, although Streptococci were the most abundant genus in mucosal tissues (hard palate 44%, oral mucosa 65%, and keratinized gingiva 66%), they represented 12–23% of the total genera detected in the other sites, including tongue (12%), supragingival (13%) and subgingival plaque (15%), and saliva, both without (15%) and with rinsing (23%). *Neisseria*, *Prevotella*, and *Haemophilus* genera were also highly prevalent in most sites, ranging from 6 to 29% of the total bacteria detected. Bacteria belonging to the *Rothia* genus, Gram-positive round-rod shaped bacteria belonging to the *Actinomycetaceae* family, were also quite abundant, ranging from 4 to 24% in all sites except for keratinized gingiva, where it was scarce. As expected, anaerobes (*Actinomyces*, *Veillonella*, *Fusobacterium*) were generally less prevalent and particularly represented in subgingival plaque specimens. *Simonsiella* was almost exclusively detected in the hard palate, confirming previous reports obtained by 16S rRNA NGS [17]. At the species level, *Streptococcus mitis* was the most prevalent (9.5% of the total detected species), followed by S. *oralis*, *salivarius*, and *sanguinis* (in order of abundance). The cariogenic *S. mutans* was detected in low amounts, representing 0.003% of the total species. *Haemophilus parainfluenzae* was the most abundant species belonging to the *Haemophilus* genus (relative abundance 11.8%), followed by *H. haemolyticus* (0.4%) and *influenzae* (0.3%). *Prevotella melaninogenica*, *Neisseria subflava* and *Rothia dentocariosa* were the most prevalent species of the respective genera, representing respectively 7.8, 5.2 and 4.9% of the total species identified in the oral microbiome by WGS analysis. In supplementary **Table S****1** all the detected species and their relative abundance values are reported.

Among eukaryotic microbes, mycetes were detected belonging to the *Saccharomicetales*, mainly to the *Candida* genus, identified as *C. albicans* species. Fungi represented, however, a very low percentage of the total microbiome (0.004%), and were almost exclusively detected in the hard palate, supra-gingival plaque and oral rinse specimens, likely due to very low prevalence in other sites, below the threshold of detectability by our method. No protozoa were detectable in any of the specimens from the study group.

Instead, a fair amount of viruses was detectable in most of the mucosal sampled sites and supragingival plaque, representing 0.03% of the total microbial normalized counts. Most of the detected viruses were bacteriophages belonging to the *Caudovirales* order, including *Siphoviridae*, *Myoviridae* and *Podoviridae* families. In addition, eukaryotic viruses of the *Herpesvirales* order, *Herpesviridae* family, were detected (0.0006% of the total normalized counts). Streptococcal phages were, however, the most prevalent viruses, above all *Streptococcus* phage K13, followed by *Aggregatibacter* phage S1249, and many other *Streptococcus* viruses. In addition, bacteriophages targeting *Haemophilus*, *Mannheimia*, *Klebsiella*, and *Actinomyces* were also detected (Fig. 1d).

Interestingly, the virome composition of oral rinse reflected quite precisely that detected in the specific sampled sites (hard and soft tissues), at least for the most prevalent species/genera. By contrast, saliva specimens were not equally representative of the whole virome present in the oral cavity, being characterized by very different abundance values of the most prevalent oral viruses.

**Fig. 2:**
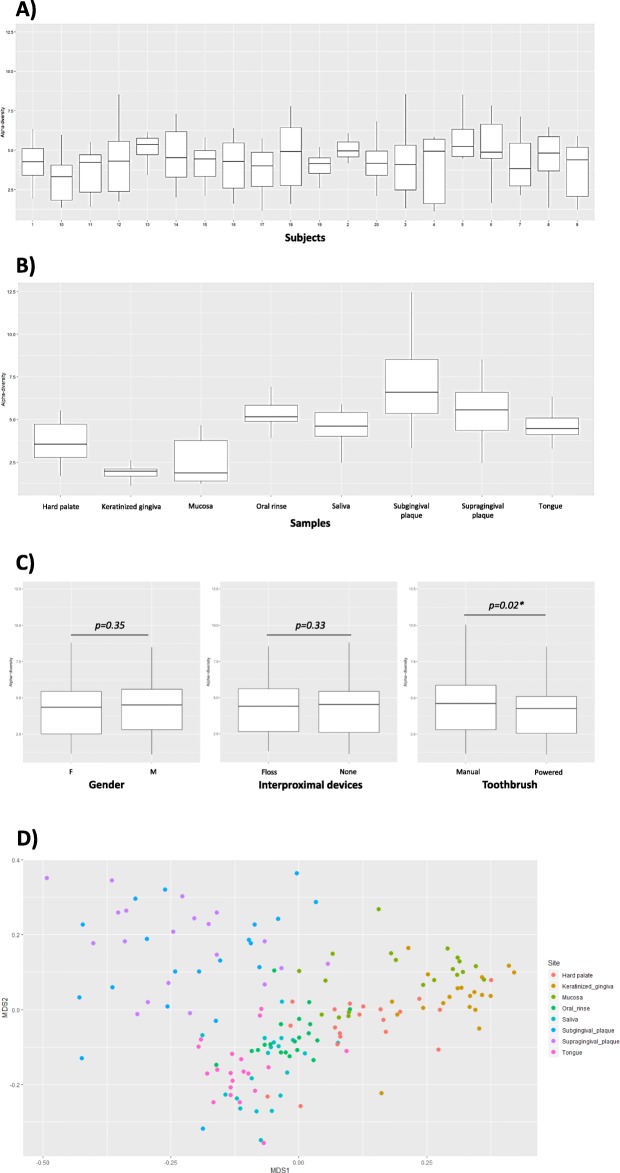
Alpha and beta-diversity analysis of HOM in collected samples. **a** Alpha-diversity values in each enrolled study participant. **b** Alpha-diversity values in all samples grouped for the specific sampling site. **c** Alpha-diversity values in sub-groups of study participants, subdivided for gender (left panel) (M, male; F, female), use of an interproximal device (tooth floss or none), and use of manual or powered toothbrush. Median line, interquartile range, and min-max values for each box-plot are shown. The *p* value as detected by Student’s *t* test is also shown in panel **c**. **d** Beta-diversity, as detected by Weighted Unifrac analysis, in the different site-specific samples from all the study participants. Multidimensional scaling (MDS) analysis of Weighted Unifrac similarity index is shown

The analysis of microbiome alpha-diversity values in the twenty study participants, performed by the measurement of the Shannon index, evidenced, as expected, an appreciable inter-individual difference between subjects (Fig. 2a), with alpha values ranging from 3.22 to 5.37. However, all the study participants clustered in one class only, and no statistically significant differences were observed among subjects, as judged by Anova test (*p* = 0.876), suggesting that the microbiome of the healthy subject (HOM) has common recognizable characteristics. On the contrary, Shannon indices of the microbiomes derived from different oral sites highlighted the high diversity of the individual microbial niches in the oral cavity, showing alpha values ranging from 1.89 to 7.03, according to which six different classes were recognized (Fig. 2b). The six different recognized clusters differed from each other in a statistically significant way (*p* < 0.0001) and included subgingival plaque (alpha value = 7.03), supragingival plaque and oral rinse (alpha values 4.56 and 5.29 respectively), tongue and saliva (respective alpha values 4.62 and 4.56), hard palate (alpha value = 3.69), mucosa (alpha value = 2.46), and keratinized gingiva (showing the lowest alpha-diversity value = 1.89).

Also, to assess the eventual presence of HOM variations related to those parameters, we analyzed the alpha-diversity subdividing the study participants for gender, use of manual versus powered toothbrush, or use of hygiene devices (i.e. floss). As summarized in Fig. 2c, the analysis of alpha diversity evidenced no differences between investigated groups except for those using a manual or powered toothbrush, that clustered in different classes (*p* = 0.02).

Similarly to what observed by alpha-diversity analysis, beta-diversity evaluation, by Weighted Unifrac, identified discernible patterns corresponding to the different oral micro-habitats, confirming the significant difference among site-specific microbiomes (Fig. 2d). Elevated similarities were observed between supra- and sub-gingival plaque, oral mucosa and keratinized gingiva, saliva and oral rinse, saliva and tongue surface.

**Fig. 3:**
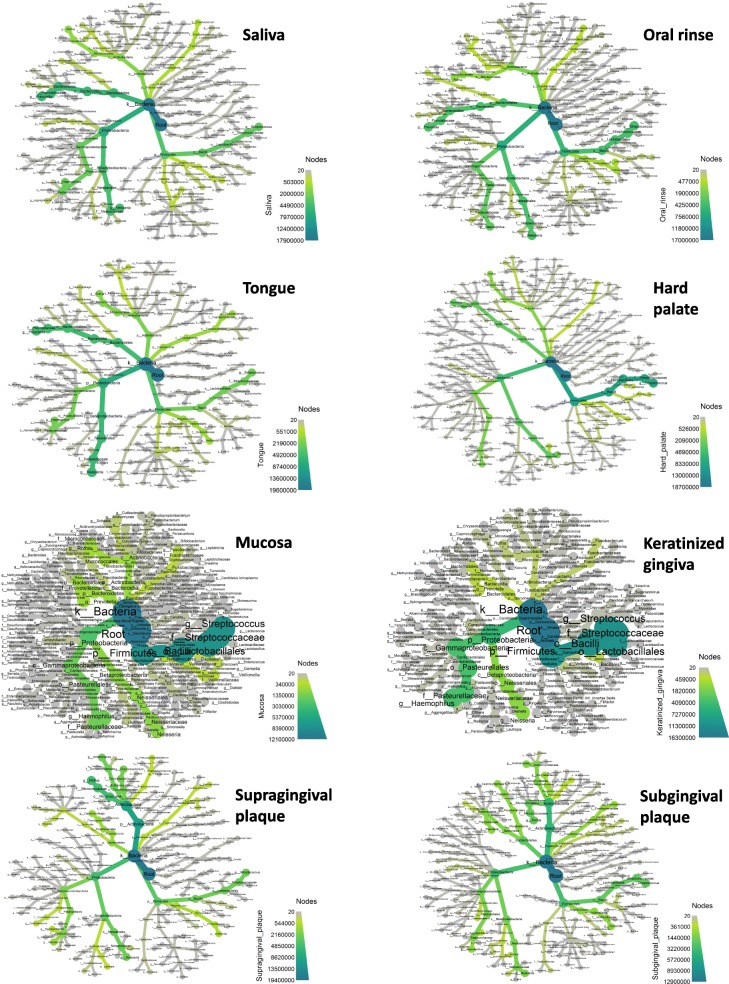
Heat-tree representation of the relative abundance of the detected, subdivided for sampled sites. Phyla, classes, orders, families, genera, and species are represented. Node label, taxon name; node size, number of operational taxonomic units (OTUs); node color, abundance of the indicated phylum/class/family/genus/species (from grey to green, as reported in the color scale)

Since beta-diversity only provides a summary of the differences between samples in a single number, but cannot show in detail why the samples are different, the results were additionally elaborated and represented as heat-trees, to display differences in the abundance of each taxon in the analyzed samples. Such a graphical representation of diversity in the different oral microhabitats, summarized in Fig. 3, highlights the differences among collected oral sites.

To assess whether it was possible to find out a microbial signature identifying the different micro-habitat of the oral cavity, data were also analyzed by PAM (Prediction Analysis of Microarrays), which is a statistical technique for class prediction from gene expression data using nearest shrunken centroids [41]. The method of nearest shrunken centroids identifies subsets of genes that best characterize each class. The technique is general and can be used in many other classification problems, thus we applied it for the analysis of our data, to identify the genera that best define the microbiome site-specificity, allowing to recognize the site of origin based on a statistical prediction.

**Fig. 4:**
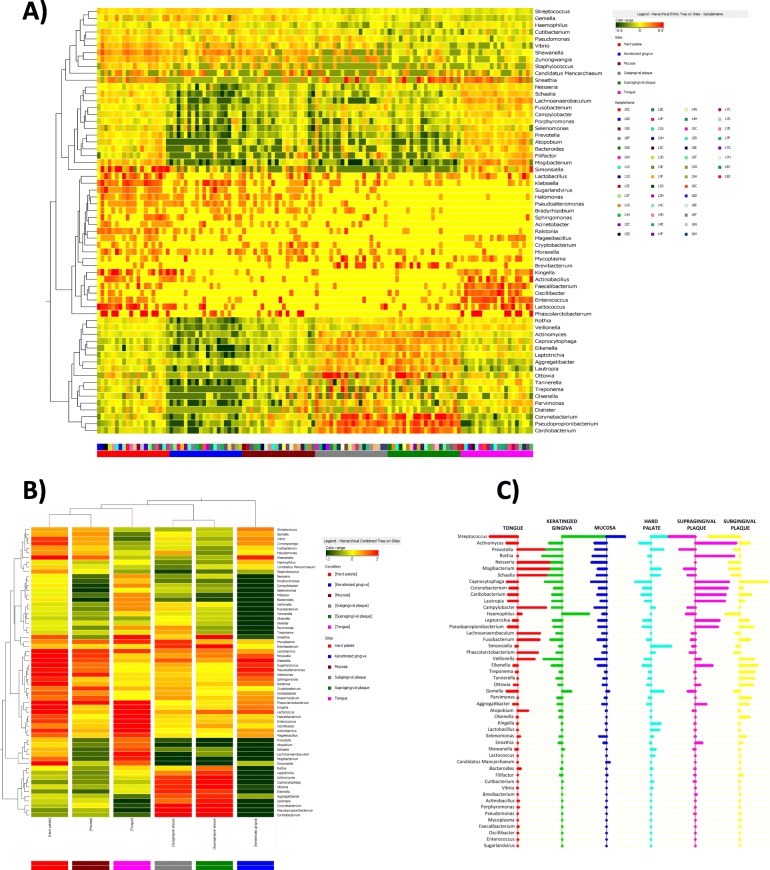
Heatmap representing the distribution of the most 62 representative HOM microbial genera. **a** Results obtained in the different sampled oral sites from all the study participants (excluding saliva samples). **b** Mean prevalence values of the most representative 62 genera in each collection site (excluding saliva). Hierarchical legends are also shown for both panels. **c** Microbial genera best defining the site-specificity of the oral microbiome, as detected by PAM analysis

The analysis showed that 62 genera resulted particularly useful in identifying the collection site of oral specimens. Figure 4a summarizes the genera usefully recognized for this analysis, as detected in each individual sampled site in the 20 enrolled subjects. Grouping together the results obtained in each collection site, it was evident that such 62 genera were differently present in the different sites, thus allowing to recognize specific microbiomes for each microhabitat. The different prevalence of the indicated genera in each microhabitat was even more evident by grouping in each collection site the values obtained as a mean of the 20 subjects (Fig. 4b).

Such genera were analyzed by the Prediction Analysis for Microarray (PAM) for class prediction, using the nearest shrunken centroid methodology. Briefly, the method computes a standardized centroid for each class and compares each new sample to each of these class centroids, allowing to assess whether a parameter can predict the membership class.

The results of PAM analysis, summarized in Fig. 4c, showed that 49 microbial genera, out of the 62 identified as the more characteristic of each specific oral site, indeed allowed in most cases the recognition of the specific oral site of origin of the collected sample.

Namely, by using such method, 77% of samples were correctly classified (Table 3). A larger cohort would likely provide a more precise classification. However, samples from the tongue, oral mucosa, supragingival plaque, and keratinized gingiva were predictable 80–90% of times, whereas hard palate and subgingival plaque were less predictable by these microbial genera (55–60%). Particularly the hard palate resulted difficult to classify with respect to other sites, and subgingival plaque were difficultly distinguishable from supragingival plaque in our study group.

**Table 3:** Cross-tabulation of true (rows) versus predicted (columns) classes for PAM fit

ORAL SITE	Tongue	Keratinized gingiva	Oral mucosa	Hard palate	Supragingival plaque	Subgingival plaque
**Tongue**	18	0	0	2	0	0
**Keratinized gingiva**	0	16	4	0	0	0
**Oral mucosa**	0	2	18	0	0	0
**Hard palate**	3	3	2	12	0	0
**Supragingival plaque**	0	0	1	0	17	2
**Subgingival plaque**	1	0	2	0	6	11

The PAM classifier performance was characterized by the following parameters: sensitivity 0.55–0.9, specificity 0.91–0.99, positive predictive value (PPV) 0.667–0.923, and negative predictive value (NPV) 0.916–0.98 (Table 4).

**Table 4:** Performance of PAM classifier during cross-validation

ORAL SITE	Sensitivity	Specificity	PPV	NPV
**Tongue**	0.9	0.96	0.82	0.98
**Keratinized gingiva**	0.8	0.95	0.76	0.96
**Oral mucosa**	0.9	0.91	0.67	0.98
**Hard palate**	0.6	0.99	0.92	0.93
**Supragingival plaque**	0.85	0.94	0.74	0.97
**Subgingival plaque**	0.55	0.98	0.85	0.92

*PPV* positive predictive value, *NPV* negative predictive value

**Fig. 5:**
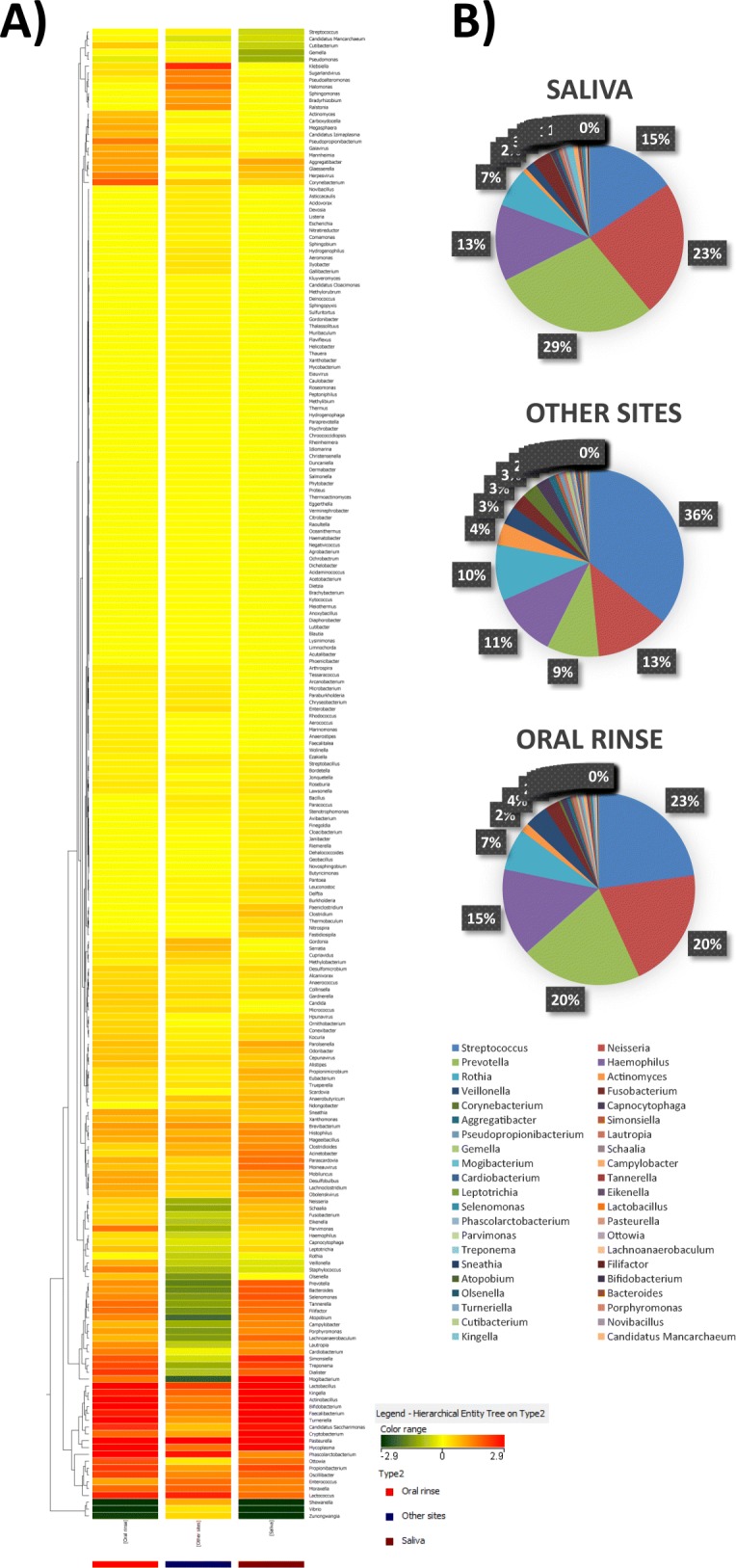
Distribution and relative abundance of microbial genera in saliva, oral rinse and other sites. **a** Comparison of mean values detected in saliva, oral rinse, and other sites (hard and soft tissues). Hierarchical legend is also shown. **b** Comparison of mean relative abundance values detected in saliva, oral rinse and other sites (hard and soft tissues)

Another point that we wanted to address in this study was to understand if a unique sample of saliva or saliva after rinsing (oral rinse) could be representative of the whole oral microbiome, especially for future comparative studies. To this purpose, the heat-map obtained by grouping the mean values from all enrolled subjects in saliva or oral rinse was compared with the mean values obtained putting together all the values derived from the other sampled sites. The results of comparative analysis, summarized in Fig. 5a, showed that saliva and oral rinse had very similar microbiomes, but that important differences could be evidenced, showing that oral rinse was more closely representing all the genera/species detected by site-specific sampling, rather than saliva. In particular, several highly prevalent and less represented bacterial genera (including *Streptococcus*, *Candidatus*, *Cutibacterium*, *Gemella*, *Pseudomonas*, *Actinomyces*, *Pseudopropionibacterium*, *Aggregatibacter*, *Corynebacterium*, *Staphylococcus*, *Veillonella*, *Parvimonas*, and *Micrococcus*) were more abundantly detected in oral rinse compared to saliva. Furthermore, the difference was especially evident for more infrequent genera/species, such as *Candida* spp. and the oral viruses belonging to the oral virome, which were more detectable in oral rinse versus saliva samples (see also Fig. 3 for the virome comparison).

Consistently with this, the comparative analysis of the relative abundance of microbial genera confirmed a higher similarity between oral rinse and the site-specific samples (*p* = 0.98, by ANOVA test), compared to what observed in saliva versus other sites (*p* = 0.70) (Fig. 5b).

### Resistome analysis

Based on the results obtained by WGS, showing that oral rinse samples can be considered more representative, compared to saliva, of the whole oral microbiome, HOM resistome was characterized on oral rinse specimens. Resistance genes were identified and quantified by a specific qPCR microarray, simultaneously detecting 84 resistance genes directed against different drug classes, including aminoglycosides, β-lactams, erythromycin, fluoroquinolones, macrolide-lincosamide-streptogramin B, tetracycline, and vancomycin.

**Fig. 6:**
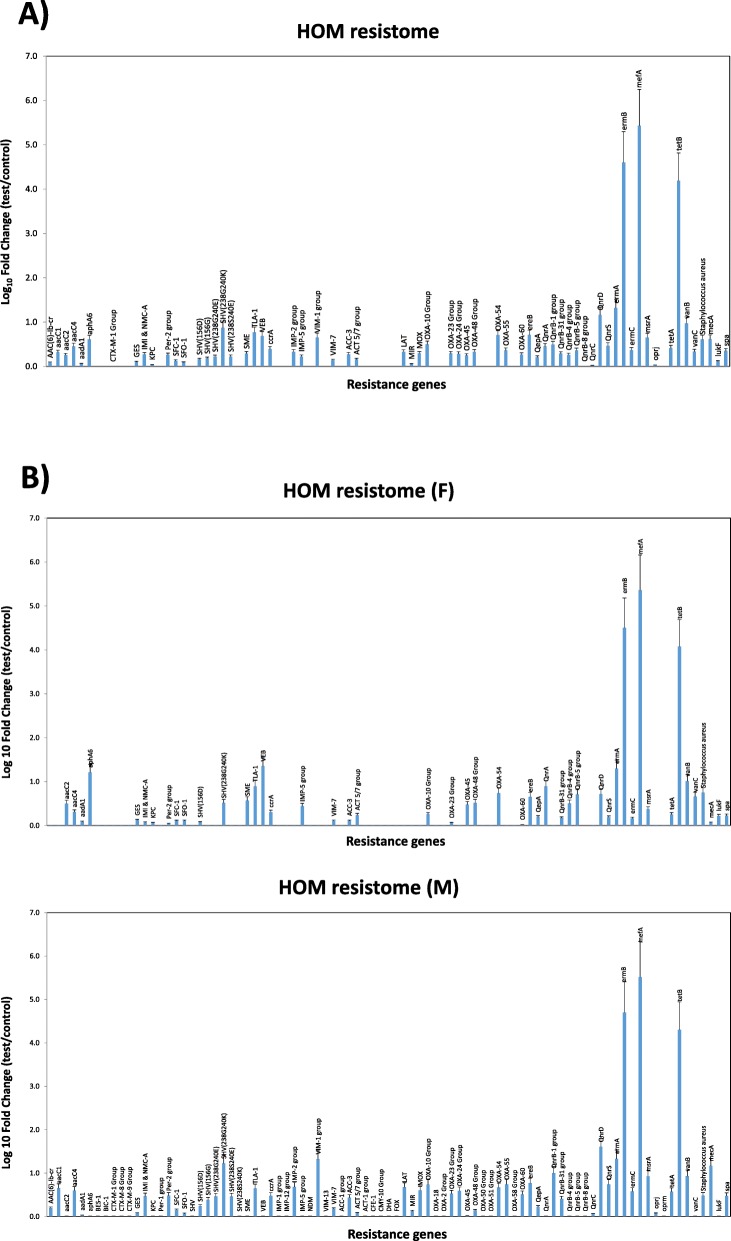
Characterization of the HOM resistome, as detected by qPCR microarray analysis. **a** HOM resistome in the whole study group. **b** Comparison of HOM resistomes of female (F) and male (M) study participants. I both panels the results are expressed as the mean value ± SD of Log_10_ fold change of oral rinse samples compared to negative controls, for each indicated resistance gene

Data obtained by microarray, summarized in Fig. 6a, showed that HOM harbors several resistance genes, some of which are highly represented.

In particular, *mefA*, *ermB*, and *tetB* resulted especially prevalent, with respective Log_10_ fold change values corresponding to 5.43, 4.60 and 4.19 compared to negative controls. Notably, *mefA* (macrolide efflux protein A) and *ermB* were reported to be prevalent in *viridans* groups streptococci (VGS), alone or in combination [42]. They confer to streptococci macrolide (M) and macrolide-lincosamide-streptogramin B (MLS_B_) phenotypes [43, 44], i.e. the resistance to macrolides including erythromycin, lincosamides (such as clindamycin) and analogs of streptogramin B. The resistance functions as an efflux pump to regulate intracellular macrolide levels, and macrolide resistance genes are usually located in mobile elements such as transposons, favoring their transmission between different bacteria. Similarly, oral biofilm was observed to be a reservoir of tetracycline resistance genes, and *tetB* was recently identified in strains of *S. oralis* [45]. The resistance to tetracycline is mediated via an active efflux pump. Our data confirm such previous observations and highlight the presence of *tetB* in Gram-positive bacteria as another step in the dissemination of antibiotic resistance genes.

Other resistance genes were present in the HOM (Log_10_ fold change value > 1.0), although they were less represented compared to macrolide-resistance genes. They included *ermA*, also conferring as *ermB* the resistance against macrolides, frequently found in oral Gram-positive bacteria [46], and *SHV*-group resistance genes, conferring resistance against beta-lactams and present in many Gram-positive bacteria including streptococci. Of note, *QnrD* gene (quinolone-resistance determinant D), a plasmid-mediated quinolone resistance typically present in Gram-negative bacteria, was also detected in HOM. This resistance gene, first reported in a human clinical isolate of Salmonella in China [47], was then detected in *Proteus mirabilis* and *Morganella morganii* in Europe, and subsequently spread in other *Enterobacteriaceae* [48, 49].

Some differences were observed between female and male cohorts of participants (Fig. 6b). Although most of them were not statistically significant, especially those related to the most prevalent resistance genes (*ermB*, *mefA*, *tetB*), significant differences (*p* < 0.01) were found in other less represented genes, including *aac* genes, *SHV*-group, *VIM-1* group, *Qnr* group, *msrA*, *vanC* and *mecA*. In particular, *aac1*, *SHV*-group, *VIM-1* group, *Qnr*-group, *msrA* and *mecA* genes were more prevalent in males than in females, whereas the contrary was observed for *aac2* and *vanC*.

## Discussion

Compared to culture-based methods, the new molecular 16S rRNA gene NGS techniques have provided a substantially deeper picture of the complexity of the bacterial component of the oral microbiome, highlighting the significant differences between the healthy oral microbiome (HOM) and local or systemic disease conditions. Depending on the oral site and individuals, many health-associated species have been identified by using the 16S rRNA NGS approach, showing the common presence of bacterial genera belonging to *Streptococcus, Granulicatella, Neisseria, Haemophilus, Corynebacterium, Rothia, Actinomyces, Prevotella, Capnocytophaga, Porphyromonas* and *Fusobacterium* [17, 20]. These studies suggested that most oral taxa found in unrelated healthy individuals were similar, and supported the concept of a healthy core microbiome, although other reports evidenced a significant subject-to-subject variation [21].

However, while allowing identification of bacteria at the level of species, NGS does not provide sufficient detail to identify bacteria at the sub-species level, and, even more importantly, it cannot simultaneously evidence the important non-bacterial components of the oral microbiome, including eukaryotic microbes (fungi, protozoa) and viruses. Consistently, the reports on HOM have been almost exclusively restricted to the study of bacteriome, and there are very few reports on the concomitant presence of other microorganisms.

More recently, the implementation of the whole-genome sequencing (WGS) techniques, have allowed overcoming such limitations, although it was used quite sporadically and not in a high number of samples from the oral cavity [23, 35, 36], also due to the high costs of such type of analysis. The advantage of WGS over NGS consists in providing quantitative strain-level data, covering simultaneously all the classes of microorganisms, including eukaryotes and viruses.

However, reports focused in depth on HOM, performing a comprehensive analysis of an adequate number of subjects, are still missing, and there is the need for studies providing a high-resolution high-throughput characterization of the healthy microbial communities in the oral microhabitats, to be used as a reference for future comparative studies. Similarly, besides, population studies on the HOM resistome are also missing, although the great importance to collect data on the prevalence and type of drug resistance of the oral microbes, due to the growing AMR concern worldwide.

The present study was therefore aimed to obtain a site-specific map of the HOM in the young adult healthy subject by using WGS as the method of analysis. To this purpose, the oral microbiomes of twenty healthy individuals (10 males and 10 females) were systematically analyzed by WGS and qPCR microarray for HOM and HOM-resistome characterization, both on site-specific oral samples (hard and soft tissues), and on saliva and oral rinse specimens.

The results showed the presence of over 200 microbial genera in the HOM of healthy individuals, evidencing a high diversity between oral microhabitats, whereas no statistically significant differences were observed between enrolled subjects, and thus strongly supporting the notion that a core HOM can be recognized and used as a reference for eubiosis definition. Of note, no statistically significant differences were found related to gender or use of hygiene devices (floss), whereas a diverse alpha-diversity value was detected in the group using manual versus powered toothbrush, suggesting that manual hygiene allows a more variable composition of HOM compared with a powered one.

Streptococci represented the most abundant genus in mucosal tissues (44–66% in the hard palate, oral mucosa, and keratinized gingiva), but they represented 12–23% of the total genera in the other sites (tongue, supragingival and subgingival plaque, saliva, and oral rinse). At the species level, *Streptococcus mitis* was the most prevalent, followed by S. *oralis*, *salivarius*, and *sanguinis*. The cariogenic *S. mutans* was detected in low amounts (0.003% of the total species). *Neisseria*, *Prevotella*, *Rothia*, and *Haemophilus* genera were also highly prevalent in most sites (representing 4 29% of the total bacteria). *Haemophilus parainfluenzae* was the most abundant species belonging to the *Haemophilus* genus (relative abundance 11.8%), followed by *H. haemolyticus* and *H. influenzae*. *Prevotella melaninogenica*, *Neisseria subflava* and *Rothia dentocariosa* were the most prevalent species of the respective genera. Anaerobes (*Actinomyces*, *Veillonella*, *Fusobacterium*) were particularly represented in subgingival plaque specimens, and *Simonsiella* was exclusively detected in the hard palate, confirming previous reports obtained by 16S rRNA NGS [17].

Mycetes, mostly *Candida* and other *Saccharomicetales*, were also detected by WGS, strengthening the notion of a mycome being part of the HOM in eubiosis conditions. Fungi represented however a small portion of the total microbiome (0.004%), present only in the hard palate, supra-gingival plaque, and oral rinse specimens, likely due to the nature of the study population, including exclusively young healthy subjects with good oral hygiene. Consistently with this, and contrarily to what reported in some studies, no protozoa were detected in any of the enrolled subjects. Also this finding might be due to the type of our study population, which was very homogeneous as to the age and general health conditions, thus rendering unlikely the oral colonization by protozoa above the threshold of detectability (10 copies per sample).

By contrast, viruses were detectable at high frequency in HOM of our study group, strengthening the notion of a core virome in HOM. In particular, viruses were detected in mucosal samples and supragingival plaque, representing 0.03% of the total microbial normalized counts. The oral virome resulted particularly rich and complex, including several phages directed against the most prevalent bacterial species in the mouth (*Siphoviridae*, *Myoviridae* and *Podoviridae* families), and human viruses of the *Herpesviridae* family. Among phages *Streptococcus* phage K13 was the most prevalent, followed by *Aggregatibacter* phage S1249, and many other *Streptococcus* viruses. In addition, bacteriophages targeting *Haemophilus*, *Mannheimia*, *Klebsiella*, and *Actinomyces* were also identified.

Of note, 49 microbial genera, including also Streptococci viruses, were detected that can predict quite precisely the origin of oral samples, and might, therefore, be used to classify the putative origin of clinical oral specimens and/or as markers of the site-specific HOMs. Only hard palate resulted hardly classifiable by these genera, and subgingival plaque was difficultly distinguishable from supragingival plaque. This might be due to the features of our study group, since in the healthy young adult, in the absence of gingivitis or periodontitis signs, the composition of subgingival plaque might be quite similar to that of supragingival plaque.

Previous works discussed profusely on how different oral biogeographic niches, the influence of geography, climate and ethnicity may influence the composition of the oral microbiome, as most data deriving from the HMP analysis of the 16S rRNA gene were actually obtained in subjects from a limited geographical area [50–52]. Indeed, also our data derive from a limited geographical location (all the enrolled subjects were from Northern Italy), that could potentially hamper the generalizability of the results. However, previous reports obtained in different populations provided similar results, as at the genus level over 200 genera were found in the oral microbiota, with hard palate showing the lowest total richness, while the gingival plaque the highest total richness [53]. In the few reports using WGS approaches, *Firmicutes*, *Actinobacteria*, *Bacteroidetes*, *Fusobacteria*, and *Proteobacteria*, accounted for 80–95% of the entire oral microbiome, similarly to what detected in our study [54]. Available WGS data for saliva and subgingival microbiome, showed more than 175 bacterial species in saliva, in two subjects [36], and significant differences of subgingival plaque in healthy or diabetic subjects [55]. These data highlight the extreme effectiveness of WGS in the detection of even low-abundant taxon, and support its potential in the definition of a core HOM and its fine characterization.

Although the limited number of subjects enrolled in our study, the sample size and power calculation, performed using a web application including preset control data from the Human Microbiome Project, evidenced a resulting power of 0.96 for twenty subjects, supporting the reliability of the collected results toward the definition of a core HOM.

The major factors driving inter-subjects differences in our study were the subject gender and the use of manual/powdered toothbrush and interproximal devices. Among these, data showed that only the type of toothbrush was significantly associated with diverse microbial composition. Importantly, our study wanted to investigate also the drug resistance features of the microorganisms composing the HOM in healthy conditions. Resistome studies have previously been performed in very few subjects, [37, 38], and a comprehensive picture of HOM resistome is still missing. Our analysis, performed by qPCR microarray, evidenced the presence of a high proportion of resistant strains, harboring resistance (R) genes most conferring resistance against macrolides, lincosamides, streptogramin, tetracycline, and quinolones. Although this kind of analysis did not allow identification of the species of origin of detected resistance genes, likely they might include streptococci, which are very abundant in the oral cavity, and Gram-negative oral bacteria, that would be interesting to identify in future studies.

Overall, macrolide-lincosamide-streptogramin (MLS) and tetracyclin R genes appeared the more abundant in the HOM, confirming data obtained in the gut [56], and suggesting that their broad distribution might be correlated with the antibiotic use in farm food animals [57]. These data, obtained by microarray, represent the first characterization of HOM from this point of view, and evidence the prevalence of important drug-resistance genes directed against common antibiotics and potentially exchangeable between bacteria, due to their plasmid nature. In addition, resistome data, contrarily to what observed for microbiome diversity, evidenced a statistically significant difference in the prevalence of some R genes between female and male study participants. In particular, male subjects harbored higher amounts of *aac*, *SHV-*group, *VIM-1* groups, *Qnr*, *msrA*, *vanC* and *mecA* R genes, whereas *aac2* and *vanC* genes were more present in the female gender. However, although these data are of interest, especially in the light of the so-called gender medicine, they should be confirmed in a larger set of subjects, as the low number of subjects enrolled in our study represents an important limitation to draw general conclusions. Due to the relevance of the resistome picture, especially in view of the global antimicrobial resistance concern, our data suggest that a more profound analysis in future NGS or WGS studies could be highlight important aspects, useful in monitoring the spread of resistant strains and its eventual gender-related differences. Overall, our WGS and microarray approach allowed to characterize the peculiar microbial population of each oral micro-habitat, evidencing differences related to gender and hygiene habits of the study participants, as well as the possibility to use the oral rinse to describe efficiently the whole oral microbiome.

The findings provide for the first time a comprehensive critical baseline for future studies interpreting microbiome-related diseases and might be usefully used for future comparative studies.

## Conclusions

Our study provides for the first time a comprehensive and detailed picture of the healthy oral microbiome as determined by WGS analysis, including also the drug-resistance features of the bacterial component, identifying the eubiosis conditions in the young adult. Importantly, as no high inter-individual differences were detected (even between genders), this gives a reference for future studies. Our data might be of relevance for all the studies focusing on oral microbiome and its variations, to identify and characterize the dysbiosis conditions occurring during various types of disease, both at the oral and systemic level.

## Methods

### Ethic statement

Recruitment of study participants was performed according to the protocol approved by the local Ethic Committee: approval document n. 684/2018/Oss/UniFe, approved on January 23rd 2019, by the Ethic Committee “Area Vasta Emilia Centro della Regione Emilia-Romagna” (CE-AVEC).

### Aim, design and setting of the study

A Single-Arm, Single-Visit observational study was performed to characterize the oral microbiome and resistome in the young healthy subject. The study was performed according to the current standards for observational clinical trials. Each enrolled subject underwent the following protocol: Visit 1 - SCREENING and ENROLLMENT: consent secured; screening and entry into the study; baseline data collection, including full periodontal charting (full-mouth pocket depth, PD; bleeding on probing, BOP; and O’Leary plaque control report, O’Leary PCR); identification of sampling sites; sampling collection. Systemic and oral health of recruited subjects were assessed by careful anamnestic interview and extra-intra oral examination.

Sample size and statistical power were calculated by using the web application available at https://fedematt.shinyapps.io/shinyMB (https://www.ncbi.nlm.nih.gov/pubmed/27153704), which has control datasets preset on Human Microbiome Project protocols (including saliva). Briefly, by using HMP saliva samples data as ‘Control group’ and data from the highly prevalent and less represented bacterial genera in a preliminary set of our samples (including *Streptococcus*, *Candidatus*, *Cutibacterium*, *Gemella*, *Pseudomonas*, *Actinomyces*, *Pseudopropionibacterium*, *Aggregatibacter*, *Corynebacterium*, *Staphylococcus*, *Veillonella*, *Parvimonas*, and *Micrococcus*) as ‘Case group’, we compared the statistical power of different sample sizes ranging from 5 to 100 samples, using the Wilcoxon-Mann-Whitney test. For a number of 20 samples in both groups the resulting power was 0.96.

### Study participants

Twenty healthy subjects, including 10 males and 10 females, aged from 18 to 30 years (mean age 24.7, range 21–30), all from Northern Italy, were enrolled for the study.

Inclusion criteria were: aged 18–30 years; good general health (free of systemic diseases such as diabetes, HIV infection or genetic disorder, ongoing malignant disease of any type that could interfere with the evaluation of the study objectives); good oral health (free of oral pathologies such as leukoplakia, erythroplakia, OLP); availability for the 6-month duration of the study for an assigned subject; signed Informed Consent Form.

Exclusion criteria included: presence of heart diseases or blood pressure alteration that requires a medication; presence of renal, hepatic, gastrointestinal disease that requires a medication; presence of diabetes; presence of any sexually transmitted disease (STD), HIV, HCV infection; presence of a genetic disorder that could interfere with the evaluation of the study objectives; chronic obstructive pulmonary disease and asthma; presence of any neoplastic lesion or cancer or paraneoplastic syndrome; current radiotherapy or chemotherapy; pregnant or lactating women; more than 8 missing teeth, with missing teeth, accounted for by third molar extractions, teeth extracted for orthodontic purposes, teeth extracted because of trauma, or congenitally missing teeth; presence of orthodontic appliances; tumors or significant pathology of the soft or hard tissues of the oral cavity (such as LPO, erythroplakia, leukoplakia, candidiasis); chronic dry mouth, as assessed through a questioning of the subject by an experienced clinician; clinically meaningful halitosis as determined by organoleptic assessment by an experienced clinician; diagnosis of periodontitis; untreated carious lesions or oral abscesses; current or past (within 3 months before enrolment) assumption of medications that may influence periodontal conditions and/or interfere with healing following periodontal treatment (i.e., corticosteroids, calcium channel blockers, systemic antibiotics).

### Collection of clinical specimens

Oral micro-habitats were sampled similarly to what described in the Human Microbiome Project protocol (Manual of Procedures for Human Microbiome Project, Core Microbiome Sampling, Protocol A, HMP Protocol # 07–001, Version Number: 12.0, 29 Jul 2010) [58].

The specimens included saliva (with and without rinsing), four soft tissues (tongue, hard palate, buccal mucosa, keratinized gingiva), and two hard tissues (supra- and sub-gingival plaque).

For saliva collection, the subject was asked to let the saliva collect in the mouth for at least 1 min, then asked to drool it into a sterile 50 ml collection tube to collect a minimum volume of 2–5 ml.

For saliva after rinsing, study participants rinsed their mouth with 15 mL sterile phosphate buffered saline (PBS) for 1 min, and expectorated the contents of the mouth into a 50 mL centrifuge tube.

Soft tissue sites were sampled using sterile sample collection rayon Swabs (Copan, Brescia, Italy). Immediately after swabbing, each swab was put in a 1.5 ml sterile microtube containing 0.5 ml of sterile phosphate buffered solution (PBS), braking the swab rod to allow closing the microtube. Sampled sites included: tongue dorsum (1 cm^2^ area at the center of the tongue, rubbed for 5 s), hard palate (the entire area, rubbed for 10 s), buccal mucosa (the entire area of both sides, rubbed for 10 s each), keratinized (attached) gingiva (the maxillary anterior attached gingiva, rubbed for 10 s).

Sampling of hard sites was performed from six index teeth. Briefly, each index tooth was isolated with cotton rolls and dried with a gentle stream of air from an air-water syringe. With a Gracey curette, all of the supragingival plaque from the mesial surface of the selected index tooth was collected. The curette tip was immersed in 0.5 ml sterile PBS contained in a sterile 1.5 ml tube for 4–5 s and wiped off on the inside edge of the collection tube. The supragingival plaque samples from the six teeth were pooled in one tube.

For subgingival plaque collection, any residual supragingival plaque was removed before collection from the subgingival area. The subgingival plaque was sampled from the mesiobuccal surface of the selected six index teeth with a sterile Gracey curette, and collected in sterile tubes as described for supragingival specimens. Any specimens with significant amounts of blood, based on clinical judgment (i.e., if flooding occurs) was discharged. Similarly, the subgingival plaque samples from six teeth were pooled in one tube.

All collected specimens were immediately refrigerated (2–8 °C) and transported to the laboratory for analysis. All clinical samples were processed within 1 h.

### Sample DNA extraction

Collected samples were vortexed (3 × 30 s), and each collected sample was centrifuged at 14000 x g for 10 min at 4 °C to pelletize the corpuscular part and separate it from extracellular soluble components (supernatant). The cell pellets were frozen at − 80 °C until use. Total DNA was then extracted from the pellets by the Exgene Cell SV Kit (Gene All, Tema Ricerca, Bologna, Italy), following manufacturer’s instructions modified by introducing a pre-lysis step with 5 mg/ml of lysozyme to obtain optimal lysis of Gram-positive bacteria. Briefly, samples were incubated for 30 min at 37 °C, then DNA was purified in a final elution volume of 100 μl [59]. The obtained DNA was checked and quantified by nanodrop spectrophotometric (Thermo Scientific, Milan,Italy) reading at 260/280 nm. The quality of extracted DNA was checked by amplifying 10 ng of extracted DNA by two PCRs targeting human β-actin and bacterial 16S rRNA gene (panbacterial PCR, *panB*), as previously described [60–62].

### Library preparation and sequencing

Following verification of DNA amplificability, 100 ng of total extracted DNA were analyzed by whole genome sequencing (WGS) by the NGS Service of the University of Ferrara (Department of Morphology, Surgery and Experimental Medicine, University of Ferrara), who carried out library preparation, sequencing and taxonomic analysis.

Raw sequencing data and bioinformatics analyses have been deposited in the European Nucleotide Archive (ENA) website (accession number PRJEB36291).

Briefly, WGS libraries were prepared using NEBNext® Fast DNA Fragmentation and Library Prep Kit for Ion TorrentTM (ThermoFisher Scientific, Milan, Italy), following the manufacturer’s protocol. Samples were then sequenced by using the Ion Gene Studio S5 System (ThermoFisher Scientific, Milan, Italy).

Taxonomic assignment has been performed using Kraken2 (Pubmed ID: 24580807) and a database consisting of archea, bacteria, fungi, protozoa, and viruses.

### Microarray analyses

The resistome of HOM population was characterized by real time quantitative PCR (qPCR) microarray performed on total DNA extracted from oral rinse specimens. Briefly, 1 μg of total DNA was analyzed by the Antibiotic Resistance Genes qPCR microarray (Cat. no. 330261 BAID-1901ZRA, Qiagen, Hilden, Germany) as previously described [62, 63], simultaneously detecting and quantifying 84 resistance genes directed against different drug classes, including aminoglycosides, β-lactams, erythromycin, fluoroquinolones, macrolide-lincosamide-streptogramin B, tetracyclines, and vancomycin. Raw microarray data have been deposited in the European Nucleotide Archive (ENA) website (accession number PRJEB36291).

### Statistical analyses

Statistical analyses have been performed with Agilent GeneSpring GX v11.5 software (Agilent Technologies, Santa Clara, CA, USA) and R (R 2019, R Core Team, available as a free software at https://www.r-project.org/). Data were expressed as relative abundance of each taxonomic unit at genus or species level. The null hypothesis was tested by Kruskal-Wallis test. Alpha- and beta-diversity were used to describe the microbiome diversity between sampled sites and subjects. Alpha-diversity was obtained by measuring the Shannon H′ diversity index. Beta-diversity was evaluated by Weighted Unifrac index. PAM (Prediction Analysis of Microarrays), based on nearest shrunken centroids, was also used for data analysis [41]. The method is freely available from the url http://www-stat.stanford.edu/˜tibs/PAM. A *p* value < 0.05 was considered significant.

## Supplementary information

**Additional file 1: Table S1.** Species detected in the oral microbiome by WGS analysis.

## Data Availability

Raw sequencing data have been deposited in the European Nucleotide Archive (ENA) website (accession number PRJEB36291).

## Abbreviations

NGS: Next generation sequencing
WGS: Whole genome sequencing
HOM: Human oral microbiome
PAM: Prediction Analysis for Microarray
HMP: Human Microbiome Project
qPCR: Real-time quantitative PCR
