# Supplementary material for: Defining the oral microbiome by whole-genome sequencing and resistome analysis: the complexity of the healthy picture
Source: BMC Microbiol. 2020 May 18;20:120. doi: 10.1186/s12866-020-01801-y (PMC7236360; doi:10.1186/s12866-020-01801-y)
Supplement: Supplementary file 1 — Additional file 1: Table S1. Species detected in the oral microbiome by WGS analysis. [file 12866_2020_1801_MOESM1_ESM.docx]

**Table S1. Species detected in the oral microbiome by WGS analysis**

| **Genus** | ***Species*** | **Genome N.** |
| --- | --- | --- |
|  |  |  |
| Acinetobacter | *Acinetobacter johnsonii* | 10 |
|  | *Acinetobacter junii* | 10 |
|  | *Acinetobacter lwoffii* | 50 |
|  | *Acinetobacter ursingii* | 20 |
| Actinobacillus | *Actinobacillus pleuropneumoniae* | 190 |
|  | *Actinobacillus suis* | 10 |
| Actinomyces | *Actinomyces hongkongensis* | 36280 |
|  | *Actinomyces oris* | 341890 |
|  | *Actinomyces pacaensis* | 50470 |
|  | *Actinomyces sp. 299* | 2620 |
|  | *Actinomyces sp. Chiba101* | 510 |
|  | *Actinomyces sp. oral taxon 171* | 529200 |
|  | *Actinomyces sp. oral taxon 414* | 9120 |
|  | *Actinomyces sp. oral taxon 848* | 73470 |
|  | *Actinomyces sp. oral taxon 897* | 5340 |
|  | *Actinomyces viscosus* | 10300 |
| Aeromonas | *Aeromonas hydrophila* | 20 |
| Aggregatibacter | *Aggregatibacter actinomycetemcomitans* | 3980 |
|  | *Aggregatibacter aphrophilus* | 173090 |
|  | *Aggregatibacter phage S1249* | 2960 |
|  | *Aggregatibacter segnis* | 42550 |
| Alistipes | *Alistipes shahii* | 150 |
| Anaerobutyricum | *Anaerobutyricum hallii* | 200 |
| Anaerococcus | *Anaerococcus mediterraneensis* | 150 |
| Anaerolineaceae | *Anaerolineaceae bacterium oral taxon 439* | 30 |
| Arcanobacterium | *Arcanobacterium haemolyticum* | 10 |
| Asticcacaulis | *Asticcacaulis excentricus* | 30 |
| Atopobium | *Atopobium parvulum* | 11340 |
| Avibacterium | *Avibacterium paragallinarum* | 30 |
| Bacillus | *Bacillus thermoamylovorans* | 20 |
|  | *Bacillus thermocopriae* | 20 |
| Bacteroides | *Bacteroides dorei* | 1860 |
|  | *Bacteroides fragilis* | 9050 |
|  | *Bacteroides heparinolyticus* | 640 |
|  | *Bacteroides salanitronis* | 10 |
|  | *Bacteroides thetaiotaomicron* | 540 |
|  | *Bacteroides zoogleoformans* | 420 |
| Bifidobacterium | *Bifidobacterium breve* | 10 |
|  | *Bifidobacterium dentium* | 50 |
|  | *Bifidobacterium gallinarum* | 10 |
|  | *Bifidobacterium longum* | 190 |
| Bradyrhizobium | *Bradyrhizobium icense* | 20 |
|  | *Bradyrhizobium sp. BTAi1* | 40 |
|  | *Bradyrhizobium sp. CCBAU 51670* | 10 |
|  | *Bradyrhizobium sp. CCBAU 51778* | 10 |
|  | *Bradyrhizobium sp. SK17* | 20 |
| Brevibacterum | *Brevibacterium aurantiacum* | 810 |
|  | *Brevibacterium linens* | 20 |
|  | *Brevibacterium sp. CS2* | 20 |
| Burkholderia | *Burkholderia cenocepacia* | 10 |
|  | *Burkholderia contaminans* | 20 |
| Butyricimonas | *Butyricimonas faecalis* | 10 |
| Campylobacter | *Campylobacter concisus* | 169060 |
|  | *Campylobacter curvus* | 830 |
|  | *Campylobacter gracilis* | 12190 |
|  | *Campylobacter hominis* | 150 |
|  | *Campylobacter jejuni* | 130 |
|  | *Campylobacter showae* | 5490 |
|  |  |  |
| Candidatus | *Candidatus Gracilibacteria bacterium HOT-871* | 1830 |
|  | *TM7 phylum sp. oral taxon 488* | 6750 |
|  | *TM7 phylum sp. oral taxon 955* | 550 |
|  | *TM7 phylum sp. oral taxon 957* | 2750 |
| Capnocytophaga | *Capnocytophaga gingivalis* | 175720 |
|  | *Capnocytophaga haemolytica* | 21670 |
|  | *Capnocytophaga leadbetteri* | 80860 |
|  | *Capnocytophaga ochracea* | 13110 |
|  | *Capnocytophaga sp. ChDC OS43* | 121550 |
|  | *Capnocytophaga sp. oral taxon 323* | 211970 |
|  | *Capnocytophaga sp. oral taxon 864* | 16940 |
|  | *Capnocytophaga sp. oral taxon 878* | 5020 |
|  | *Capnocytophaga sputigena* | 171070 |
|  | *Capnocytophaga stomatis* | 40 |
| Cardiobacterium | *Cardiobacterium hominis* | 112310 |
| Caulobacter | *Caulobacter sp. FWC26* | 20 |
| Chroococcidiopsis | *Chroococcidiopsis thermalis* | 20 |
| Chryseobacterium | *Chryseobacterium shandongense* | 10 |
|  | *Chryseobacterium taklimakanense* | 10 |
| Clostridiales | *Clostridiales bacterium CCNA10* | 10 |
|  | *Clostridioides difficile* | 260 |
|  | *Clostridium cellulosi* | 20 |
| Collinsella | *Collinsella aerofaciens* | 110 |
| Comamonas | *Comamonas testosteroni* | 20 |
| Corynebacterium | *Corynebacterium argentoratense* | 150 |
|  | *Corynebacterium atypicum* | 20 |
|  | *Corynebacterium aurimucosum* | 10 |
|  | *Corynebacterium deserti* | 410 |
|  | *Corynebacterium diphtheriae* | 240 |
|  | *Corynebacterium glaucum* | 40 |
|  | *Corynebacterium glutamicum* | 10 |
|  | *Corynebacterium jeikeium* | 160 |
|  | *Corynebacterium matruchotii* | 1218590 |
|  | *Corynebacterium mycetoides* | 30 |
|  | *Corynebacterium renale* | 40 |
|  | *Corynebacterium simulans* | 410 |
|  | *Corynebacterium sp. NML98-0116* | 60 |
|  | *Corynebacterium stationis* | 40 |
|  | *Corynebacterium striatum* | 400 |
|  | *Corynebacterium urealyticum* | 120 |
|  | *Corynebacterium ureicelerivorans* | 150 |
|  | *Corynebacterium vitaeruminis* | 100 |
| Cryptobacterium | *Cryptobacterium curtum* | 790 |
| Cupriavidus | *Cupriavidus metallidurans* | 230 |
| Cutibacterium | *Cutibacterium acnes* | 7100 |
|  | *Cutibacterium avidum* | 40 |
|  | *Cutibacterium granulosum* | 40 |
| Desulfobulbus | *Desulfobulbus oralis* | 70 |
| Devosia | *Devosia sp. H5989* | 10 |
| Dialister | *Dialister pneumosintes* | 3810 |
| Diaphorobacter | *Diaphorobacter polyhydroxybutyrativorans* | 10 |
| Eikenella | *Eikenella corrodens* | 71100 |
| Enterococcus | *Enterococcus cecorum* | 60 |
|  | *Enterococcus faecalis* | 110 |
|  | *Enterococcus faecium* | 1570 |
| Escherichia | *Escherichia coli* | 130 |
| Eubacterium | *Eubacterium minutum* | 220 |
|  | *Eubacterium rectale* | 30 |
|  | *Eubacterium sulci* | 157900 |
| Ezakiella | *Ezakiella massiliensis* | 20 |
| Faecalibacterium | *Faecalibacterium prausnitzii* | 630 |
| Faecalitalea | *Faecalitalea cylindroides* | 30 |
| Fastidiosipila | *Fastidiosipila sanguinis* | 60 |
| Filifactor | *Filifactor alocis* | 13560 |
| Finegoldia | *Finegoldia magna* | 10 |
| Flaviflexus | *Flaviflexus salsibiostraticola* | 50 |
| Fusobacterium | *Fusobacterium hwasookii* | 41720 |
|  | *Fusobacterium necrophorum* | 6660 |
|  | *Fusobacterium nucleatum* | 223850 |
|  | *Fusobacterium periodonticum* | 1295650 |
|  | *Fusobacterium sp. oral taxon 203* | 8770 |
| Gallibacterium | *Gallibacterium anatis* | 80 |
| Gardnerella | *Gardnerella vaginalis* | 110 |
| Gemella | *Gemella haemolysans* | 114130 |
|  | *Gemella morbillorum* | 40070 |
|  | *Gemella sp. ND 6198* | 650 |
|  | *Gemella sp. oral taxon 928* | 8190 |
| Glaesserella | *Glaesserella sp. 15-184* | 30 |
| Gordonia | *Gordonia bronchialis* | 700 |
|  | *Gordonia phthalatica* | 2730 |
| Haemophilus | *Haemophilus aegyptius* | 710 |
|  | *Haemophilus ducreyi* | 550 |
|  | *Haemophilus haemolyticus* | 136620 |
|  | *Haemophilus influenzae* | 96060 |
|  | *Haemophilus parainfluenzae* | 4359790 |
|  | *Haemophilus pittmaniae* | 25230 |
|  | *Haemophilus sp. oral taxon 036* | 207490 |
| Halomonas | *Halomonas campaniensis* | 40 |
|  | *Halomonas venusta* | 40 |
| Helicobacter | *Helicobacter typhlonius* | 10 |
| Histophilu | *Histophilus somni* | 490 |
| Hydrogenophilus | *Hydrogenophilus thermoluteolus* | 20 |
| Idiomarina | *Idiomarina loihiensis* | 20 |
| Janibacter | *Janibacter indicus* | 20 |
| Kingella | *Kingella kingae* | 320 |
| Klebsiella | *Klebsiella oxytoca* | 930 |
|  | *Klebsiella pneumoniae* | 190 |
| Kocuria | *Kocuria palustris* | 70 |
| Lachnoanaerobaculum | *Lachnoanaerobaculum umeaense* | 15710 |
|  | *Lachnoclostridium phocaeense* | 360 |
| Lachnospiraceae | *Lachnospiraceae bacterium oral taxon 500* | 2660 |
| Lactobacillus | *Lactobacillus amylovorus* | 30 |
|  | *Lactobacillus buchneri* | 20 |
|  | *Lactobacillus crispatus* | 80 |
|  | *Lactobacillus curvatus* | 10 |
|  | *Lactobacillus delbrueckii* | 30 |
|  | *Lactobacillus fermentum* | 950 |
|  | *Lactobacillus gasseri* | 250 |
|  | *Lactobacillus helveticus* | 460 |
|  | *Lactobacillus johnsonii* | 30 |
|  | *Lactobacillus rhamnosus* | 30 |
|  | *Lactobacillus salivarius* | 50 |
|  | *Lactococcus lactis* | 2050 |
|  | *Lactococcus raffinolactis* | 20 |
| Lautropia | *Lautropia mirabilis* | 117520 |
| Lawsonella | *Lawsonella clevelandensis* | 150 |
| Leptotrichia | *Leptotrichia buccalis* | 16890 |
|  | *Leptotrichia sp. oral taxon 212* | 71890 |
|  | *Leptotrichia sp. oral taxon 498* | 7890 |
|  | *Leptotrichia sp. oral taxon 847* | 2880 |
| Listeria | *Listeria monocytogenes* | 10 |
| Lutibacter | *Lutibacter sp. LPB0138* | 10 |
| Lysinimonas | *Lysinimonas sp. 2DFWR-13* | 10 |
| Mageeibacillus | *Mageeibacillus indolicus* | 420 |
| Mannheimia | *Mannheimia haemolytica* | 420 |
|  | *Mannheimia sp. USDA-ARS-USMARC-1261* | 10 |
| Methylobacterium | *Methylobacterium aquaticum* | 40 |
|  | *Methylobacterium sp. C1* | 10 |
|  | *Methylorubrum populi* | 20 |
| Microbacterium | *Microbacterium aurum* | 70 |
| Micrococcus | *Micrococcus luteus* | 200 |
| Mobiluncus | *Mobiluncus curtisii* | 220 |
| Mogibacterium | *Mogibacterium diversum* | 119330 |
|  | *Mogibacterium pumilum* | 1120 |
| Moraxella | *Moraxella catarrhalis* | 210 |
|  | *Moraxella osloensis* | 280 |
| Mycobacterium | *Mycobacterium avium* | 10 |
|  | *Mycobacterium paragordonae* | 10 |
| Mycoplasma | *Mycoplasma orale* | 600 |
|  | *Mycoplasma pullorum* | 100 |
|  | *Mycoplasma salivarium* | 840 |
| Ndongobacter | *Ndongobacter massiliensis* | 130 |
| Neisseria | *Neisseria animalis* | 20 |
|  | *Neisseria cinerea* | 14740 |
|  | *Neisseria elongata* | 370790 |
|  | *Neisseria flavescens* | 85090 |
|  | *Neisseria gonorrhoeae* | 29530 |
|  | *Neisseria lactamica* | 119130 |
|  | *Neisseria meningitidis* | 101720 |
|  | *Neisseria mucosa* | 492010 |
|  | *Neisseria polysaccharea* | 40250 |
|  | *Neisseria sicca* | 904950 |
|  | *Neisseria sp. KEM232* | 5060 |
|  | *Neisseria sp. oral taxon 014* | 228160 |
|  | *Neisseria subflava* | 1932200 |
| Nitratireductor | *Nitratireductor sp. OM-1* | 20 |
| Nitrospira | *Nitrospira defluvii* | 30 |
| Olsenella | *Olsenella sp. oral taxon 807* | 6710 |
|  | *Olsenella uli* | 420 |
|  | *Olsenella umbonata* | 30 |
| Ornithobacterium | *Ornithobacterium rhinotracheale* | 10 |
| Oscillibacter | *Oscillibacter sp. PEA192* | 1060 |
| Ottowia | *Ottowia sp. oral taxon 894* | 3540 |
| Pantoea | *Pantoea ananatis* | 10 |
|  | *Pantoea sp. PSNIH2* | 190 |
| Paraburkholderia | *Paraburkholderia phytofirmans* | 40 |
| Paracoccus | *Paracoccus yeei* | 10 |
| Paraprevotella | *Paraprevotella xylaniphila* | 90 |
| Parascardovia | *Parascardovia denticolens* | 120 |
| Parvimonas | *Parvimonas micra* | 30780 |
| Pasteurella | *Pasteurella multocida* | 10250 |
|  | *Pasteurellaceae bacterium NI1060* | 310 |
| Phoenicibacter | *Phoenicibacter congonensis* | 10 |
| Porphyromonas | *Porphyromonas asaccharolytica* | 220 |
|  | *Porphyromonas cangingivalis* | 20 |
|  | *Porphyromonas crevioricanis* | 330 |
|  | *Porphyromonas gingivalis* | 6060 |
| Prevotella | *Prevotella dentalis* | 800 |
|  | *Prevotella denticola* | 108350 |
|  | *Prevotella enoeca* | 31230 |
|  | *Prevotella fusca* | 53300 |
|  | *Prevotella intermedia* | 183840 |
|  | *Prevotella jejuni* | 1784480 |
|  | *Prevotella melaninogenica* | 2873420 |
|  | *Prevotella oris* | 113280 |
|  | *Prevotella scopos* | 136100 |
|  | *Prevotella sp. oral taxon 299* | 135240 |
| Propionibacterium | *Propionibacterium acidifaciens* | 150 |
|  | *Propionimicrobium sp. Marseille-P3275* | 50 |
| Proteus | *Proteus mirabilis* | 40 |
| Pseudomonas | *Pseudomonas antarctica* | 40 |
|  | *Pseudomonas fluorescens* | 230 |
|  | *Pseudomonas mendocina* | 70 |
|  | *Pseudomonas putida* | 2330 |
|  | *Pseudomonas sp. LG1D9* | 20 |
|  | *Pseudomonas sp. MYb193* | 160 |
|  | *Pseudomonas sp. phDV1* | 40 |
|  | *Pseudomonas stutzeri* | 70 |
| Pseudopropionibacterium | *Pseudopropionibacterium propionicum* | 315580 |
| Psychrobacter | *Psychrobacter sp. P11F6* | 10 |
| Ralstonia | *Ralstonia insidiosa* | 30 |
|  | *Ralstonia mannitolilytica* | 10 |
|  | *Ralstonia pickettii* | 240 |
| Raoultella | *Raoultella planticola* | 20 |
| Rheinheimera | *Rheinheimera sp. D18* | 10 |
| Roseburia | *Roseburia hominis* | 10 |
|  | *Roseburia intestinalis* | 20 |
| Roseomonas | *Roseomonas sp. FDAARGOS_362* | 10 |
| Rothia | *Rothia aeria* | 41730 |
|  | *Rothia dentocariosa* | 1829930 |
|  | *Rothia mucilaginosa* | 1475630 |
| Salmonella | *Salmonella enterica* | 50 |
| Scardovia | *Scardovia inopinata* | 10 |
| Schaalia | *Schaalia cardiffensis* | 450 |
|  | *Schaalia meyeri* | 830 |
|  | *Schaalia odontolytica* | 122570 |
| Selenomonas | *Selenomonas sp. oral taxon 126* | 2030 |
|  | *Selenomonas sp. oral taxon 136* | 16650 |
|  | *Selenomonas sp. oral taxon 478* | 33670 |
|  | *Selenomonas sp. oral taxon 920* | 4300 |
|  | *Selenomonas sputigena* | 12360 |
| Serratia | *Serratia marcescens* | 40 |
| Shewanella | *Shewanella algae* | 5520 |
|  | *Shewanella sp. FDAARGOS_354* | 10 |
| Simonsiella | *Simonsiella muelleri* | 270700 |
| Sphingobium | *Sphingobium hydrophobicum* | 10 |
|  | *Sphingobium sp. MI1205* | 20 |
|  | *Sphingobium yanoikuyae* | 30 |
| Sphingomonas | *Sphingomonas melonis* | 30 |
|  | *Sphingomonas paucimobilis* | 40 |
|  | *Sphingomonas sanxanigenens* | 30 |
|  | *Sphingomonas sp. AAP5* | 30 |
|  | *Sphingomonas sp. FARSPH* | 230 |
|  | *Sphingomonas sp. NIC1* | 10 |
| Staphylococcus | *Staphylococcus aureus* | 860 |
|  | *Staphylococcus capitis* | 140 |
|  | *Staphylococcus epidermidis* | 730 |
|  | *Staphylococcus haemolyticus* | 20 |
|  | *Staphylococcus saprophyticus* | 50 |
|  | *Staphylococcus warneri* | 70 |
| Streptobacillus | *Streptobacillus moniliformis* | 90 |
| Streptococcus | *Streptococcus agalactiae* | 57080 |
|  | *Streptococcus anginosus* | 174470 |
|  | *Streptococcus australis* | 101400 |
|  | *Streptococcus canis* | 10 |
|  | *Streptococcus constellatus* | 1090 |
|  | *Streptococcus cristatus* | 72310 |
|  | *Streptococcus dysgalactiae* | 14460 |
|  | *Streptococcus equi* | 450 |
|  | *Streptococcus equinus* | 92580 |
|  | *Streptococcus ferus* | 70 |
|  | *Streptococcus gallolyticus* | 1470 |
|  | *Streptococcus gordonii* | 146460 |
|  | *Streptococcus intermedius* | 29340 |
|  | *Streptococcus lutetiensis* | 50520 |
|  | *Streptococcus milleri* | 4030 |
|  | *Streptococcus mitis* | 3500250 |
|  | *Streptococcus mutans* | 960 |
|  | *Streptococcus oralis* | 1510510 |
|  | *Streptococcus parasanguinis* | 107890 |
|  | *Streptococcus parauberis* | 10 |
|  | *Streptococcus pluranimalium* | 220 |
|  | *Streptococcus pneumoniae* | 463980 |
|  | *Streptococcus porcinus* | 3510 |
|  | *Streptococcus pseudopneumoniae* | 163690 |
|  | *Streptococcus pyogenes* | 21000 |
|  | *Streptococcus salivarius* | 1112990 |
|  | *Streptococcus sanguinis* | 400230 |
|  | *Streptococcus sobrinus* | 950 |
|  | *Streptococcus sp. A12* | 126920 |
|  | *Streptococcus sp. ChDC B345* | 671750 |
|  | *Streptococcus sp. FDAARGOS_192* | 38190 |
|  | *Streptococcus sp. FDAARGOS_522* | 140110 |
|  | *Streptococcus sp. HSISM1* | 139980 |
|  | *Streptococcus sp. HSISS1* | 5230 |
|  | *Streptococcus sp. HSISS2* | 7420 |
|  | *Streptococcus sp. HSISS3* | 7570 |
|  | *Streptococcus sp. I-G2* | 104970 |
|  | *Streptococcus sp. I-P16* | 175430 |
|  | *Streptococcus sp. JS71* | 118310 |
|  | *Streptococcus sp. KCOM 2412* | 16380 |
|  | *Streptococcus sp. NPS 308* | 60210 |
|  | *Streptococcus sp. oral taxon 064* | 307880 |
|  | *Streptococcus sp. oral taxon 431* | 467840 |
|  | *Streptococcus suis* | 2400 |
|  | *Streptococcus thermophilus* | 34780 |
|  | *Streptococcus uberis* | 30 |
|  | *Streptococcus urinalis* | 210 |
|  | *Streptococcus vestibularis* | 140030 |
|  | *Streptococcus viridans* | 40920 |
|  | *Streptococcus virus 9872* | 30 |
| Tannerella | *Tannerella forsythia* | 12030 |
|  | *Tannerella sp. oral taxon HOT-286* | 99900 |
| Tessaracoccus | *Tessaracoccus sp. Marseille-P5995* | 10 |
| Thauera | *Thauera sp. MZ1T* | 10 |
| Treponema | *Treponema denticola* | 3980 |
|  | *Treponema putidum* | 220 |
|  | *Treponema sp. OMZ 838* | 6680 |
|  | *Treponema succinifaciens* | 410 |
| Trueperella | *Trueperella pyogenes* | 90 |
| Veillonella | *Veillonella dispar* | 461450 |
|  | *Veillonella parvula* | 788800 |
|  | *Veillonella rodentium* | 1750 |
| Verminephrobacter | *Verminephrobacter eiseniae* | 30 |
|  | *Vibrio cholerae* | 40 |
|  | *Vibrio fluvialis* | 2240 |
| Wolinella | *Wolinella succinogenes* | 30 |
| Xanthobacter | *Xanthobacter autotrophicus* | 50 |
| Xanthomonas | *Xanthomonas campestris* | 240 |
| Zunongwangia | *Zunongwangia profunda* | 2170 |
| Candida | *Candida* ***albicans*** | 40 |
| VIRUSES | *Herpesviridae* | 230 |
|  | *Streptococcus phage* ***5093*** | 50 |
|  | *Streptococcus* ***phage EJ-1*** | 930 |
|  | *Streptococcus phage K13* | 1290 |
|  | *Streptococcus phage PH10* | 430 |
|  | *Streptococcus phage phiARI0131-1* | 610 |
|  | *Streptococcus phage phiARI0131-2* | 240 |
|  | *Streptococcus phage phiARI0468-2* | 1320 |
|  | *Streptococcus phage phiARI0746* | 630 |
|  | *Streptococcus phage SM1* | 70 |
|  | *Streptococcus phage SpSL1* | 860 |
|  | *Streptococcus phage YMC-2011* | 30 |
|  | *Streptococcus virus Cp1* | 10 |
|  | *Streptococcus virus DT1* | 10 |
|  | *Streptococcus virus phiAbc2* | 60 |
|  | *Klebsiella virus IME260* | 70 |
|  | *Klebsiella virus Sugarland* | 40 |
